# Glioma stem cells-derived exosomal miR-26a promotes angiogenesis of microvessel endothelial cells in glioma

**DOI:** 10.1186/s13046-019-1181-4

**Published:** 2019-05-17

**Authors:** Zhi-Fei Wang, Fan Liao, Hao Wu, Jin Dai

**Affiliations:** Department of Neurosurgery, The Third Xiangya Hospital of Central South University, No. 138, Tongzipo Road, Changsha, 410013 Hunan Province People’s Republic of China

**Keywords:** Glioma stem cells, Exosomes, microRNA-26a, Microvessel endothelial cells, Angiogenesis, Phosphatase and tensin homolog deleted on chromosome ten, PI3K/Akt signaling pathway

## Abstract

**Background:**

Cancer stem cells (CSCs), which are involved in cancer initiation and metastasis, could potentially release exosomes that mediate cellular communication by delivering microRNAs (miRNAs). Based on the role of miR-26a in angiogenesis of glioma, our study was performed to investigate whether glioma stem cells (GSCs)-derived exosomes containing miR-26a could exert effects on angiogenesis of microvessel endothelial cells in glioma, in order to provide a new therapeutic RNA vehicle for glioma therapies.

**Methods:**

The expression of miR-26a and PTEN in glioma was quantified and the interaction among miR-26a, PTEN and PI3K/Akt signaling pathway was examined. Next, a series of gain- and loss-of function experiments were conducted to determine the role of miR-26a in angiogenesis of human brain microvascular endothelial cells (HBMECs). Subsequently, HBMECs were exposed to exosomes derived from GSCs with the gain−/loss-of-function of miR-26a. Finally, the effect of exosomal miR-26a on angiogenesis of HBMECs was assessed both in vitro and in vivo.

**Results:**

The results revealed that PTEN was down-regulated, while miR-26a was up-regulated in glioma. miR-26a activated the PI3K/Akt signaling pathway by targeting PTEN. Restored miR-26a promoted proliferation, migration, tube formation, and angiogenesis of HBMECs in vitro. In addition, GSCs-derived exosomes overexpressing miR-26a contributed to enhanced proliferation and angiogenesis of HBMECs in vitro through inhibition of PTEN. The angiogenic effects of GSCs-derived exosomes overexpressing miR-26a in vivo were consistent with the above-mentioned in vitro findings.

**Conclusion:**

Collectively, our study demonstrates that GSCs-derived exosomal miR-26a promotes angiogenesis of HBMECs, highlighting an angiogenic role of miR-26a via exosomes.

**Electronic supplementary material:**

The online version of this article (10.1186/s13046-019-1181-4) contains supplementary material, which is available to authorized users.

## Background

Glioma is the general term used to indicate all types of primary central nervous system tumors and is the most common type of primary brain tumors with poor prognosis [1, 2]. The incidence of glioma has been identified to be 4.67–5.73 per 100,000 people [3]. Despite the recent progress made in the therapeutic options for glioma, the average survival time of patients with the malignant glioma is less than 1.5 years [4]. Recurrence of the gliomas is attributed to tumor-initiating glioma stem cells (GSCs), which are essential for gliomagenesis and have been found to be intrinsically resistant to therapy [5]. As multipotent tumor-stimulating cells, GSCs exhibit stem cell features and express CD133 marker [6]. Moreover, GSCs have been demonstrated to be involved in tumor growth and tumor angiogenesis [7, 8]. Along with a number of other reasons, resistance to glioma therapies could potentially occur due to immortalized cellular growth and aberrant angiogenesis [9]. Angiogenesis is a hallmark of cancer, due to its ability to facilitate cells transforming from benign tumors to malignant ones [10]. Moreover, angiogenesis plays a significant role in the progression and development of gliomas [11]. Therefore, extensive research is required to further elucidate the effects of GSCs on angiogenesis and the underlying mechanism in human glioma.

Extracellular vesicles (EVs) that mainly contains exosomes, ectosomes, microvesicles, microparticles, apoptotic bodies along with other EV subsets, could function in the intercellular communication [12]. Exosomes are a well-studied subpopulation of small EVs [13, 14]. Exosomes could be derived from different types of cells including neurons, immune cells, stem cells and cancer cells, which are considered to be one of the primary targets of anti-cancer therapy due to their small sizes (40–100 nm) [15]. It has been well established that exosomes are capable of transferring proteins, messenger RNAs (mRNAs) and microRNAs (miRNAs) between cells [16]. miRNA transfer mediated by exosomes has been recognized as a potential approach for miRNAs to exert their effects on various biological functions in tumorigenesis and biogenesis [17]. Recent evidence has demonstrated that exosomal miRNAs have been identified as promising biomarkers for diagnosis and prognosis of glioblastoma [18, 19]. miRNAs refer to small non-coding RNA molecules, which serve as regulators of inhibition of mRNA translation or reduction of mRNA stability by binding to the 3′ untranslated region (3’UTR) of target mRNA [20, 21]. In addition, the effects of miRNAs on glioma have been demonstrated with increasing clinical implications [22]. For instance, miR-26a has been reported to be amplified in glioma tissues and play a vital role in angiogenesis of glioma [23]. Similarly, miR-4467, miR-638 and miR-6727-5p are up-regulated in the exosomes so as to affect the development of gliomas [24]. Previously, Skog et al. have demonstrated that exosomes derived from GSCs can be internalized by brain microvascular endothelial cells (MVECs), thus serving as a new option for the delivery of genetic information and proteins to recipient cells in tumor environment [25]. A number of studies have shown that phosphatase and tensin homolog deleted on chromosome ten (PTEN) is one of the most common tumor suppressor genes inactivated in various cancers [26]. PTEN regulates various cellular processes including cellular architecture, energy metabolism, proliferation and survival by mediating the PI3K-AKT-mTOR signaling pathway [26, 27]. Based on the aforementioned findings, we hypothesized that miR-26a delivered from GSCs into MVECs via exosomes could potentially provide an effective therapeutic strategy for glioma by controlling PTEN and regulating the PI3K-Akt pathway. Therefore, in this study, we isolated GSCs from glioma cells to elucidate the regulatory role of exosomal miR-26a in angiogenesis of MVECs and the molecular mechanism associated with PTEN and PI3K-Akt pathway, in an attempt to provide a theoretical foundation for glioma treatment.

## Materials and methods

### Ethics statement

This study was approved by the Ethics Committee of The Third Xiangya Hospital of Central South University and all the included patients in the study signed written consents. All animal experiments were conducted in strict accordance with the Guidelines for Care and Use of Laboratory Animals.

### Microarray analysis

Glioma microarray datasets were obtained from the Gene Expression Omnibus (GEO) database (https://www.ncbi.nlm.nih.gov/geo/), and the Limma package of R language [28] was used to identify the differentially expressed genes (DEGs) related to glioma. The Gene oncology (GO) enrichment analysis was conducted with the WebGestalt database (http://www.webgestalt.org/option.php). Besides, the DisGeNET database (http://www.disgenet.org/web/DisGeNET/menu/home) was used to predict glioma-related genes. The interaction among the DEGs was analyzed by using the STRING database (https://string-db.org/), and the signaling pathways related to PTEN was retrieved by using the Kyoto Encyclopedia of Genes and Genomes (KEGG) database (https://www.kegg.jp/kegg/pathway.html). The miRDB database (http://mirdb.org/miRDB/index.html), mirDIP database (http://ophid.utoronto.ca/mirDIP/index.jsp), microT-CDS (http://diana.imis.athena-innovation.gr/DianaTools/index.php?r=microT_CDS/index) in DIANA database, and TargetScan database (http://www.targetscan.org/vert_71/) were used to predict the miRNAs that regulate PTEN.

### Tissue collection

All fresh tissue samples were surgically resected from 46 patients with pathologically confirmed glioma between September 2015 and September 2017 at the Department of Neurosurgery, The Third Xiangya Hospital of Central South University. There were 26 males and 20 females with an mean age of 42.16 ± 13.57 years (ranging from 18 to 72 years). Among these patients, there were 13 cases of astrocytoma I stage (WHO I stage), 16 cases of astrocytoma II stage (WHO II stage), 13 cases of malignant anaplastic astrocytoma (WHO III stage), and 4 cases of glioblastoma multiforme (WHO IV stage) [29]. None of the patients included in the study had received radiotherapy or chemotherapy prior to the surgery. Subsequently, brain tissues were collected from 28 patients with acute craniocerebral injury (19 males and 9 females, 28–57 years, mean age of 42.43 ± 8.14 years) that had received treatment of intracranial decompression.

### Cell culture

The glioma cell lines, SHG-44 (astrocytoma), BT325 (glioblastoma), T98G (glioblastoma), A172 (glioblastoma), U251 (astrocytoma), normal human glial cells (HEB), and human brain microvascular endothelial cells (HBMECs) were purchased from Cell Resource Center, Shanghai Institute for Biological Sciences, Chinese Academy of Sciences (Shanghai, China). After recovery, the cells were cultured in a 5% CO_2_ and 37 °C incubator (Thermo Fisher Scientific, CA, USA) with saturated humidity with the Roswell Park Memorial Institute (RPMI) 1640 culture medium (Gibco Company, CA, USA) supplemented with 10% fetal bovine serum (FBS) (Gibco Company, CA, USA).

### Cell transfection

The HBMECs were seeded into a 12-well culture plate at a concentration of 1 × 10^5^ cells/well 1 day before transfection. When cell confluence reached 50–70%, HBMECs were transfected with miR-26a agomir, agomir-negative control (NC), miR-26a antagomir, antagomir-NC, PTEN overexpression vector and PTEN overexpression NC (PTEN-NC) using lipo2000 (11,668,027, Thermo Fisher Scientific, CA, USA). After 6 h of culture, the culture medium was renewed. And cells were collected for RNA and protein extraction after 48 h of transfection.

### Dual-luciferase reporter gene assay

The biological prediction website Targetscan.org was used to predict the binding sites of miR-26a in the PTEN sequence, and dual-luciferase reporter gene essay was performed to verify whether PTEN was a direct target gene of miR-26a. The synthesized PTEN 3’UTR gene fragment was digested by enzyme sites XhoI and BamHI and inserted into pGL3-control vector (Promega Corporation, Madison, WI, USA). The mutant (Mut) sites were designed in the supplementary sequences of seed sequences in PTEN wild type (Wt) and PTEN Mut sequences, Xho I and BamH I. The target fragments were inserted into the pGL3-control vectors using T4 DNA ligase. The pGL3-PTEN-Wt and pGL3-PTEN-Mut were co-transfected with miR-26a mimic and NC sequences of miR-26a mimic into glioma cell U251 (Cell Resource Center, Shanghai Institute for Biological Sciences, Chinese Academy of Sciences, Shanghai, China), respectively. After a 48-h transfection, the cells were collected and lysed. Dual-Luciferase® Reporter Assay System kit (Promega Corporation, Madison, WI, USA), and Luminometer TD-20/20 (E5311, Promega Corporation, Madison, WI, USA) were employed to determine the luciferase activity.

### RNA isolation and quantitation

The total RNA was extracted by using a Trizol kit (Invitrogen Inc., Carlsbad, CA, USA) and reversely transcribed into complementary DNA (cDNA). The primer sequences of miR-26a and PTEN were designed and synthetized by AOKE Biotechnology Co., Ltd. (Zhenjiang, China) (Table 1). RT-qPCR was conducted with the use of the ABI StepOnePlus real time quantitative PCR instrument (StepOnePlus, ABI Company, Oyster Bay, NY, USA). U6 was used as the internal reference of miR-26a, while glyceraldehyde-3-phosphate dehydrogenase (GAPDH) was used as the internal reference of other genes. The relative mRNA expression of the target gene was analyzed by 2^-ΔΔCt^ method, using the following formula: △△Ct = △Ct _experimental group_ - △Ct _control group_, △Ct = Ct _target gene_ - Ct _internal reference_.

**Table 1:** Primer sequences of related genes for RT-qPCR

Gene	Sequence
miR-26a	F: 5′-GCCTAACCCAAGAAGGGAAA-3′
	R: 5′-TCCCTCTCATCTGGACAACC-3′
PTEN	F: 5′-AAGAGGCCCTAGATTTCTATG-3′
	R: 5′-CAGTAGAGGAGCCGTCAAAT-3′
U6	F: 5′-AATTGGAACGATACAGAGAAGATTAGC-3′
	R: 5′-TATGGAACGCTTCACGAATTTG-3′
GAPDH	F: 5′-GAGAGACCCCACTTGCTGCCA-3′
	R: 5′-GGAAGAAGTTCCCATCGTCA-3′

*RT-qPCR* reverse transcription quantitative polymerase chain reaction, *miR-26a* microRNA-26a, *PTEN* phosphatase and tensin homolog deleted on chromosome ten, *GAPDH* glyceraldehyde-3-phosphate dehydrogenase

### Western blot analysis

The total protein was extracted by using radio-immunoprecipitation assay (RIPA) lysis buffer (BB-3209, BestBio Science, Shanghai, China). The proteins were separated using sodium dodecyl sulfate (SDS)-polyacrylamide gel electrophoresis (PAGE), and transferred onto a polyvinylidene fluoride (PVDF) membrane with constant voltage of 80 V. After the membranes were blocked for 1 h, they were incubated with diluted primary antibodies, rabbit anti-human PTEN (1: 100, ab32199; Abcam Inc.. Cambridge, MA, USA), vascular endothelial growth factor (VEGF) (1: 100, sc4570, Santa Cruz Biotechnology, Inc., Santa Cruz, CA, USA), matrix metalloproteinase (MMP)-2 (1: 100, ab37150, Abcam Inc.. Cambridge, MA, USA), MMP-9 (1: 100, ab73734, Abcam Inc.. Cambridge, MA, USA), protein kinase B (Akt) (1: 100, ab8805, Abcam Inc.. Cambridge, MA, USA), p-Akt (1: 100, ab192623, Abcam Inc.. Cambridge, MA, USA), and GAPDH (1: 100, ab9385, Abcam Inc.. Cambridge, MA, USA) (internal reference) for 1 h at 37 °C. TBST washes (5 min × 3) were followed. Horseradish peroxidase (HRP)-labeled rabbit anti-human IgG (1: 20000, ab205718, Abcam Inc.. Cambridge, MA, USA) served as the secondary antibody. The membrane was developed by enhanced chemiluminescence (ECL).

### GSC isolation and characterization

The U251 cells in logarithmic growth phase were collected in an aseptic centrifuge tube containing Neurobasal stem cell culture medium. The immunomagnetic bead sorting kit and instrument were used for the separation of CD133^+^ cells in accordance with the instructions [30]. Cells were added with immunomagnetic bead sorting solution (200 μL/10^8^ cells), and triturated into single cell suspension. Meanwhile, the CD133 antibody-beads complex was incubated together with cells (100 μL/10^5^ cells) at 4 °C for 30 min, and the sorting solution was added to rinse cells (1 mL/10^8^ cells). Following centrifugation and the removal of the cell supernatant, the cells were collected and re-suspended with the addition of sorting solution (500 μL/10^8^ cells). Afterwards, the cell suspension was added into the separation column, followed by the addition of 2 mL sorting solution for elution of CD133^+^ cells. The separated CD133^+^ and CD133^−^ cells were added with 50 μL CD133/2 (293C3)-PE antibody or 50 μL homotypic control IgG2b-PE antibody, respectively, and sorted by flow cytometry.

### GSC transfection

A day prior to transfection, the cells were incubated with Dulbecco’s modified Eagle medium (DMEM)/F12 culture medium without penicillin-streptomycin at a density of 1 × 10^5^ cells/well. When GSC confluence reached 70–90%, the cells were transfected in strict accordance with the instructions of FuGENE HD6 transfection kit (E2311, Promega Corporation, Beijing, China). The GSCs were transfected with miRNA agomir (GSC^miR-26a agomir^) and agomir NC (GSC^agomir-NC^), miRNA antagomir (GSC^miR-26a antagomir^) and antagomir NC (GSC^antagomir-NC^), respectively. The final concentration of miRNA agomir or antagomir was 100 nmol/L. After a 6-h transfection, the medium was replaced by fresh stem cell medium. After 48 h of transfection, the cells obtained were used for subsequent experiments.

### GSC exosome extraction

The transfected GSCs were seeded in the exosomes-depleted RPMI 1640 culture medium containing 10% FBS, and cultured in an incubator with 5% CO_2_ at 37 °C. After 3 days, the cell supernatant was collected and centrifugation was carried out to remove cell debris. The exosomes were extracted on the basis of the instructions of Hieff™ Quick exosome isolation kit (41201ES50, Yeasen Company, Shanghai, China). The cell supernatant and separation reagent of exosome were added into the centrifuge tube (EP tube) at a ratio of 2: 1 and incubated at 4 °C overnight. Next, the samples were centrifuged at 10000×g at 4 °C for 1–2 h, followed by the removal of the supernatant, and the collection of the precipitates (exosomes). In accordance with volume ratio of initial culture medium and resuspension (10: 1), the samples were re-suspended in phosphate buffered saline (PBS), after which 30 μL of the re-suspended exosomes were placed in an EP tube and added with the equal volume of RIPA lysis buffer, mixed, and placed on ice. The exosomes were lysed twice using microwave method for 10 s. Subsequently, protein concentration in exosomes was measured via bicinchoninic acid (BCA) quantitative kit (Beyotime Biotechnology, Jiangsu, China). The particle size distribution of exosomes was analyzed with the use of a Nanosight nanoparticle tracking analyzer (Malvern Panalytical, Malvern, UK). Flow cytometry was performed to determine exosome surface markers (CD63 and CD81). Morphology of exosomes was characterized under a transmission electron microscope (TEM) (JEM-1010, JEOL, Tokyo, Japan). Finally, the exosomes secreted from GSCs (GSCs-exosomes) were identified.

The exosomes were immediately fixed in 2.5% glutaraldehyde at 4 °C. After fixation, the exosomes were dehydrated by gradient alcohol and embedded with epoxy resin. Ultrathin sections were stained with uranyl acetate and lead citrate and observed under a TEM (JEM-1010, JEOL, Tokyo, Japan).

### Flow cytometry

One μL beads were added into the EP tube containing 300 μL morpholine-ethanesulfonate (MES) buffer, and evenly mixed. Next, the mixture were centrifuged at 1610×g for 5 min. The supernatant was discarded by using a pipette. The beads were washed and then suspended using 300 μL MES buffer. Next, GSCs-exosome suspension were incubated together with the beads at room temperature for 1 h. The EP tube was rotated at 4 °C overnight. On the following day, the EP tube was centrifuged at a low speed for 1 min, and the samples were diluted into a final concentration of 200 μmol/mL with the addition of 1 mol/L glycine. Following an incubation period of 30 min at room temperature, the samples were re-suspended with 300 μL of 3% FBS-MES buffer and incubated with antibodies anti-CD63 (FITC) (ab18235, Abcam Inc., Cambridge, MA, USA) and anti-CD81 (FITC) (ab239256, Abcam Inc., Cambridge, MA, USA) at room temperature for 40 min. Subsequently, the samples were washed twice with 300 μL of 3% FBS-MES buffer. The samples were centrifuged at 16102×g for 5 min to remove the cell supernatant. Finally, the samples were re-suspended with 250 μL PBS and subjected to flow cytometric detection.

### Co-culture of exosomes and HBMECs

The HBMECs in logarithmic growth phase were seeded into a 6-well plate of RPMI 1640 culture medium containing 5% FBS (5 × 10^5^ cells/well). Subsequently, 20 μg exosomes extracted from transfected GSCs were mixed evenly with PKH26 (RED, 1: 1000), and incubated at 37 °C for 15 min. Finally, PKH26-traced exosomes were co-cultured with green fluorescent protein (GFP)-labeled HBMECs, followed by incubation with 200 μL PBS containing 1% BSA at room temperature for 20 min. The samples were stained with 4′,6-diamidino-2-phenylindole (DAPI) to stain nuclei in blue and VECTASHIELD mounting media (Vector Labs, CA, USA). The Olympus BX61 confocal fluorescence microscope (Zeiss Meta 510, Thornwood, NY, USA) was adopted to observe internalization of exosomes by HBMECs.

### Enzyme-linked immunosorbent assay (ELISA)

A total of 50 mg of tissues were homogenized for 2 min, and centrifuged at 1610×g and 4 °C for 10 min, after which the cell supernatant was obtained. VEGF concentration in the supernatant was measured in accordance with the instructions of the ELISA kit (69–50,049, Wuhan MSK Biotechnology Co., Ltd., Wuhan, China). The absorbance was detected in microplate reader (Synergy 2, BioTek, USA) at the wavelength of 450 nm within 3 min.

### 5-ethynyl-2′-deoxyuridine (EdU) labeling

HBMECs in logarithmic growth phase that had been co-cultured with GSCs-exosomes were seeded into a 96-well plate (3 × 10^4^ cells/well). The cells were cultured with EdU culture medium (100 μL/well) for 2 h, followed by incubation with 100 μL fixative for 30 min. Subsequently, incubation was carried out in each well with 2 mg/mL glycine for 5 min and with 100 μL of 0.5% TritonX-100 in PBS for 10 min. The cells were incubated again with 1 × Apollo dyeing reaction solution without light exposure for 30 min, and incubated with 100 μL 1 × Hoechst 33342 reaction solution without light exposure for 30 min in successive. After staining, 100 μL anti-fluorescence quenching agent was added into each well. A total of 6–10 fields were selected for each well, which were observed and photographed under the fluorescence microscope.

### Transwell assay

After 48 h of transfection, the Matrigel (YB356234, Shanghai YU BO Biological Technology Co., Ltd., Shanghai, China) preserved at − 80 °C was dissolved at 4 °C overnight. The cells were then trypsinized, and prepared into cell suspension. Each well in the apical chamber was added with cell suspension (1 × 10^4^ cells/μL), and the basolateral chamber was added with 800 μL medium containing 20% FBS. Following an incubation period of 16 h at 37 °C, the Transwell chamber was fixed with formaldehyde for 10 min, and cleaned three times with water. Subsequently, the samples were stained with 0.1% crystal violet for 30 min. The cells on the surface were wiped off by cotton ball. After 24 h, the cells were observed, photographed, and counted under an inverted microscope.

### Tube formation assay

Calcein-labeled HBMECs were seeded into a 24-well plate at the density of 2 × 10^5^ cells/mL. After cells attached to the wall of well, exosomes from GSCs which were transfected with miR-26a agomir or miR-26a antagomir were incubated at 37 °C with 5% CO_2_ for 12–24 h. Tube formation was observed under the confocal microscope. The images were captured at a fixed time. The number and length of branches were measured, recorded, and statistically analyzed [31].

### Angiogenesis assay on chicken chorioallantoic membrane (CAM)

A total of 60 embryonated chicken embryos were incubated at (37.5 ± 0.5)°C with 65% humidity. On the 3rd day after incubation, the vascular network growth was examined by candling, with 48 well-developed ones enrolled. GSCs transfected with agomir-NC, miR-26a agomir, antagomir-NC, or miR-26a antagomir were injected into the chicken embryos (*n* = 12 per group). On the 7th day of incubation, the fertilized chicken embryos were disinfected with ethanol. A small window (1.5 cm × 1.5 cm) was excised at the lower right side of the embryo head to expose the CAM. The sample filter paper was placed on CAM and yolk sac membrane with less blood vessels. After incubation for 10 days in a thermostat box, the transparent adhesive tape covered on the chicken embryos was removed. The angiogenesis was observed under the microscope and photographed by a digital camera [32].

### Tumorigenicity assay in nude mice

A total of 24 BALB/c female nude mice (aged 4–6 weeks old and weighing 16–22 g) purchased from Shanghai Laboratory Animal Center, Chinese Academy of Science were randomly grouped into the GSC^agomir-NC^, GSC^miR-26a agomir^, GSC^antagomir-NC^, and GSC^miR-26a antagomir^ groups, with 6 mice in each group. Subsequently, the nude mice were injected with the transfected GSCs. The single cell suspension was subcutaneously injected into the back of the nude mice using a disposable sterile syringe (4 × 10^6^ cells/mL). The tumor volume was calculated as V = W^2^ × L × 0.52 [33]. After 35 days, nude mice were euthanized, followed by the resection of the implanted tumors, which were fixed in 4% neutral formaldehyde overnight. After being embedded with paraffin, the samples were cut into 5 μm sections.

### Microvessel density (MVD) detection

The streptavidin-peroxidase immunohistochemical staining was performed with CD31 (1: 200) primary antibody. The primary antibody replaced with PBS in NC, and the CD31-positive sections served as positive control. Five fields under the microscope for each section were randomly selected, and 100 cells in each field were counted. The MVD value was expressed by the number of newly formed vessels (CD31 positive staining cells).

### Statistical analysis

Statistical analysis was performed by using the SPSS 21.0 software (IBM Corp. Armonk, NY, USA), with normality and homogeneity of variance tested. The data with normal distribution were expressed as mean ± standard deviation (SD). Comparisons between glioma brain tissues and control brain tissues were analyzed by using unpaired *t*-test, while comparisons among multiple groups were analyzed by using one-way analysis of variance (ANOVA) with Tukey’s post hoc test. The data at different time points were analyzed by repeated measurement ANOVA, and the correlation analysis was conducted using Pearson’s correlation analysis. *p* < 0.05 was considered as statistically significant.

## Results

### PTEN and miR-26a are involved in glioma

**Fig. 1:**
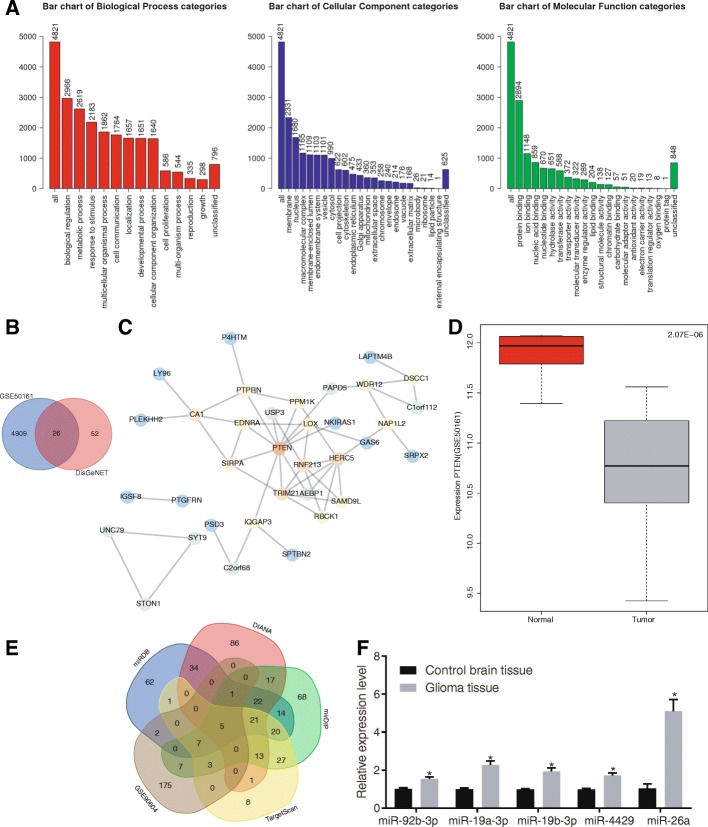
PTEN is poorly expressed in glioma and predicted to be a target of miR-26a**. a** GO enrichment analysis of DEGs in expression dataset of glioma (GSE50161); three histograms refer to the biological process (BP), cellular component (CC) and molecular function (MF) enrichment, respectively; the abscissa refers to GO entry, while the ordinate refers to the number of DEGs; **b** the intersection of DEGs and the known genes related to glioma. Two circles represent DEGs obtained from GSE50161 datasets (blue) and the known genes related to glioma obtained from DisGeNET (red), and the middle part indicates their intersection; **c** interaction analysis of glioma DEGs; each circle represents one gene, the line among circles represents the interaction among genes, and the color of circle represents the core degree of gene in the whole network; **d** PTEN expression in GSE50161 dataset; the abscissa refers to sample type, and the ordinate refers to gene expression level; the left box plot refers to the normal group, and the right box plot refers to the glioma group; **e** putative miRNAs that regulate PTEN; the intersection of putative miRNAs targeting PTEN obtained from the four databases and up-regulated miRNAs in glioma from GSE90604 dataset; the middle part refers to the intersections among five datasets; **f** the expression of miR-92b-3p, miR-19a-3p, miR-19b-3p, hsa-miR-4429 and miR-26a in control brain tissues and glioma tissues determined by RT-qPCR; the data between control brain tissues (*n* = 28) and glioma tissues (*n* = 46) are compared by unpaired-*t* test. The experiments are repeated 3 times. * *p* < 0.05, compared with the control group. DEGs, differentially expressed genes; miR/miRNA, microRNA; GO, gene oncology; PTEN, phosphatase and tensin homolog deleted on chromosome ten

The expression profile of glioma (GSE50161) was obtained from the GEO database and analyzed in order to investigate DEGs in glioma. The results found 4935 DEGs in total. GO enrichment analysis further showed that these DEGs were mainly enriched in three categories, including “biological regulation”, “membrane” and “protein binding” (Fig. 1a). In order to further clarify the DEGs related to glioma, interaction analysis and gene interaction network were performed on the top 50 DEGs. The known genes related to glioma were obtained from DisGeNET database, and the genes with scores > 0.2 were intersected with DEGs, which identified 26 intersected genes. Further gene interaction analysis was conducted on these 26 genes (Fig. 1b). Among them, PTEN, a tumor-suppressor gene, was located at the core position (Fig. 1c). Moreover, the PTEN-related signaling pathways were further retrieved from the KEGG database. Based on the findings, PTEN could potentially regulate the PI3K/Akt signaling pathway (map 05214). PTEN was down-regulated in the glioma expression dataset (Fig. 1d), indicating that PTEN may act as regulator in glioma via the PI3K/Akt signaling pathway. According to recent studies, miRNAs such as miR-29 and miR-19a-3p regulate PTEN to function as tumor promoter or suppressor [34, 35]. Four prediction websites (miRDB, mirDIP, DIANA and TargetScan database) were employed to predict the miRNAs that could regulate PTEN in order to further investigate the upstream regulation mechanism of PTEN. Meanwhile, glioma-related miRNA expression dataset GSE90604 was obtained from the GEO database and the up-regulated miRNAs were intersected with the putative miRNAs that could regulate PTEN. As shown in Fig. 1e, there were five miRNAs (hsa-miR-92b-3p, hsa-miR-19a-3p, hsa-miR-19b-3p, hsa-miR-4429 and hsa-miR-26a) in the intersection. Moreover, further analysis on these miRNAs found that compared with control brain tissues, there was a significant increase in miR-26a in glioma tissues (Fig. 1f). The above findings suggested that PTEN is lowly expressed while miR-26a highly expressed in glioma, and miR-26a might target PTEN.

### miR-26a is up-regulated in both glioma tissues and GSCs

**Fig. 2:**
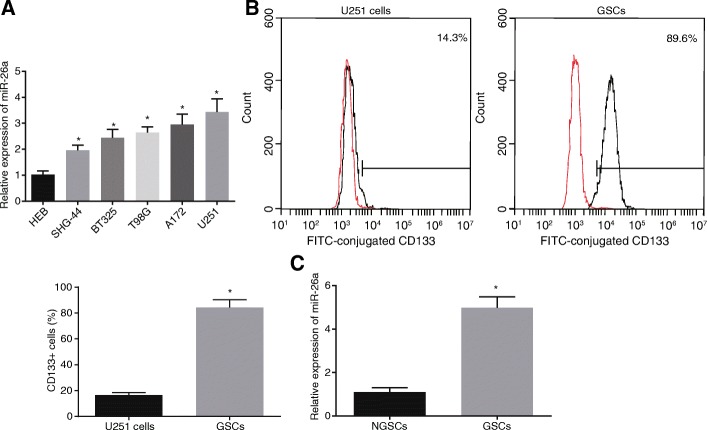
miR-26a is highly expressed in both glioma tissues and GSCs. **a** miR-26a expression in normal human glial cells (HEB) and glioma cell lines (SHG-44, BT325, T98G, A172, and U251); **b** isolation of GSCs from U251 cells using flow cytometry; **c** miR-26a expression in GSCs and NGSCs; *, *p* < 0.05, compared with the HEB (**a**), U251 cells (**b**), or NGSCs (**c**); all data were expressed as mean ± standard deviation; comparisons among multiple groups were analyzed by the one-way ANOVA; the experiment was repeated three times; ANOVA, analysis of variance; miR-26a, microRNA-26a; GSCs, glioma stem cells; NGSCs, non-glioma stem cells

To determine whether miR-26a was expressed in glioma tissues, its expression was evaluated in control brain tissues and glioma tissues by RT-qPCR, respectively (Fig. 2a). Compared with normal human glial cells (HEB), miR-26a was significantly up-regulated in five glioma cell lines, including SHG-44 (astrocytoma), BT325 (glioblastoma), T98G (glioblastoma), A172 (glioblastoma) and U251 (astrocytoma). Among those cell lines, U251 cell line presented with the highest expression of miR-26a. GSCs are a kind of multifunctional tumor initiating cells with stem cell properties [36, 37] and express CD133 marker [38, 39]. To examine whether miR-26a was also highly expressed in GSCs, flow cytometry was adopted to separate GSCs from U251 cells, and the CD133^+^ cellular expression was determined using flow cytometry/immunomagnetic beads (Fig. 2b). The successful separation of the GSCs was indicated by the evident increase observed in the CD133^+^ cellular expression. In addition, as shown in Fig. 2c, miR-26a expression was obviously elevated in GSCs. These findings suggested that miR-26a was up-regulated in both glioma tissues and GSCs.

### GSCs transfer miR-26a to HBMECs through exosomes

**Fig. 3:**
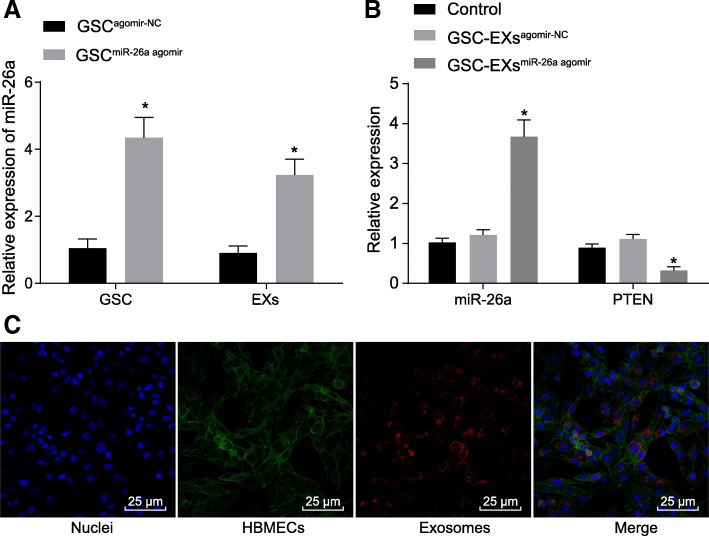
GSCs deliver miR-26a to HBMECs through exosomes. **a** miR-26a expression in GSCs transfected with miR-26a agomir and their derived exosomes using RT-qPCR; **b** miR-26a expression and PTEN expression in HBMECs co-cultured with exosomes derived from miR-26a agomir-transfected GSCs using RT-qPCR; **c** exosomes and uptake of exosomes into HBMECs observed under the confocal microscope (× 400); exosomes labeled with PKH26 was stained red; GFP-labeled HBMECs were stained green; the nucleus stained with DAPI was blue; *, *p* < 0.05, compared with the GSC^agomir-NC^ or control group; all data were expressed as mean ± standard deviation; comparisons between two groups were analyzed using unpaired *t*-test; comparisons among multiple groups were analyzed by the one-way ANOVA; the experiment was repeated three times; ANOVA, analysis of variance; RT-qPCR, reverse transcription quantitative polymerase chain reaction; miR-26a, microRNA-26a; GFP, green fluorescent protein; PTEN, phosphatase and tensin homolog deleted on chromosome ten; HBMECs, microvascular endothelial cells; GSCs, glioma stem cells; DAPI, 4′,6-diamidino-2-phenylindole; NC, negative control

The molecular mechanisms of GSCs in regulating HBMECs were investigated, the exosomes successfully isolated from GSCs (Additional file 1: Figure S1) were co-cultured with HBMECs. RT-qPCR was conducted to measure the expression of miR-26a and PTEN. Based on the results, miR-26a was up-regulated both in GSCs transfected with miR-26a agomir and exosomes derived from GSC^miR-26a agomir^ compared to that in agomir-NC-transfected GSCs and GSC^agomir-NC^ derived exosomes, respectively (Fig. 3a-b). Notably, the overexpression of miR-26a, and down-regulation of PTEN were observed in HBMECs co-cultured with GSC-exosomes in the event that miR-26a agomir was transfected into GSCs before co-culture (Fig. 3b). Notably, GSC-exosomes were observed to be internalized by HBMECs and distributed around the nucleus under the confocal microscope (Fig. 3c). Therefore, these findings led to the conclusion that GSCs transferred miR-26a to HBMECs through exosomes.

### Up-regulation of miR-26a promotes proliferation, migration and angiogenesis of HBMECs

**Fig. 4:**
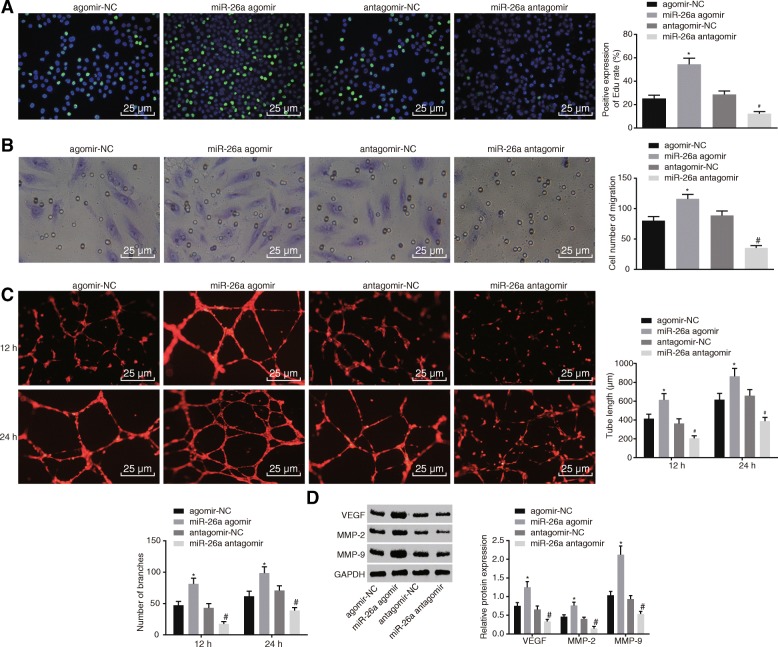
Restored miR-26a enhances HBMEC proliferation, migration, tube formation, and angiogenesis. **a** proliferation of HBMECs transfected with miR-26a agomir or miR-26a antagomir detected using EdU assay (× 400); **b** migration of HBMEC transfected with miR-26a agomir or miR-26a antagomir assessed using transwell assay (× 400); **c** tube formation of HBMECs transfected with miR-26a agomir or miR-26a antagomir (× 400); **d** protein levels of VEGF, MMP-2 and MMP-9 of HBMEC transfected with miR-26a agomir or miR-26a antagomir measured using Western blot analysis; *, *p* < 0.05, compared with the agomir-NC group; #, *p* < 0.05, compared with the antagomir-NC group; all data were expressed as mean ± standard deviation; comparisons between two groups were analyzed using unpaired *t*-test; comparisons among multiple groups were analyzed by the one-way ANOVA; the experiment was repeated three times; ANOVA, analysis of variance; miR-26a, microRNA-26a; HBMECs, human brain microvascular endothelial cells; EdU, 5-ethynyl-2′-deoxyuridine; VEGF, vascular endothelial growth factor; MMP-2, matrix metalloproteinase-2; MMP-9, matrix metalloproteinase-9; NC, negative control

To determine whether miR-26a could affect proliferation, migration and angiogenesis of HBMECs in vitro, the HBMECs were transfected with miR-26a agomir, or miR-26a antagomir. As shown in Fig. 4a-c, the overexpression of miR-26a enhanced the HBMEC proliferation, migration and tube formation. In contrast, inhibition of miR-26a expression caused the reduction in HBMEC proliferation, migration and tube formation. VEGF exerts strong effects on promoting angiogenesis when combined with corresponding receptors [40]. MMP is a type of extracellular proteolytic enzyme, which can degrade basal membrane and promote invasion and migration of malignant tumors, of which MMP-2 and MMP-9 are two primary enzymes to degrade type IV collagen [41]. The results of Western blot analysis (Fig. 4d) revealed that the up-regulation of miR-26a elevated the protein levels of VEGF, MMP-2, and MMP-9, whereas inhibition of miR-26a expression decreased protein levels of VEGF, MMP-2, and MMP-9. These findings suggested that elevated miR-26a promoted HBMEC proliferation, migration and tube formation in vitro.

### miR-26a activates the PI3K/Akt signaling pathway by down-regulating PTEN in glioma

**Fig. 5:**
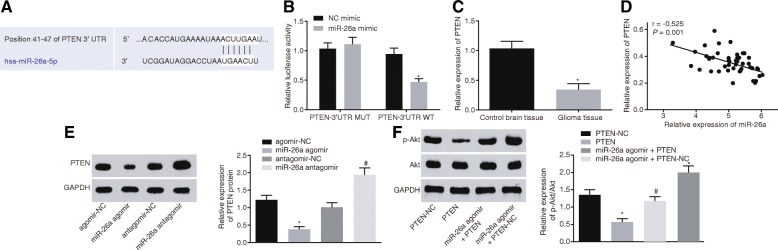
miR-26a activates the PI3K/Akt signaling pathway via down-regulation of PTEN. **a** the miR-26a binding site in PTEN 3’UTR; **b** detection of luciferase activity; **c** PTEN expression in glioma tissues and control brain tissues; **d** correlation analysis of miR-26a expression with PTEN expression; **e** PTEN protein expression in HBMECs transfected with miR-26a agomir or miR-26a antagomir; **f** Akt protein level and phosphorylation level in HBMECs transfected with PTEN overexpression vector and/or miR-26a agomir measured using Western blot analysis; *, *p* < 0.05, compared with NC mimic (**b**), control brain tissues (**c**), agomir-NC (**e**), or PTEN-NC (**f**) group; #, *p* < 0.05, compared with antagomir-NC or miR-26a agomir + PTEN-NC group; All data were expressed as mean ± standard deviation; there were 46 cases of glioma tissues and 28 cases of control brain tissues; the correlation analysis was conducted using Pearson correlation analysis; comparisons between two groups were analyzed using unpaired *t*-test; comparisons among multiple groups were analyzed by the one-way ANOVA; the experiment was repeated three times; ANOVA, analysis of variance; HBMECs, human brain microvascular endothelial cells; miR-26a, microRNA-26a; NC, negative control; PTEN, phosphatase and tensin homolog deleted on chromosome ten; Akt, protein kinase B; GAPDH, glyceraldehyde-3-phosphate dehydrogenase

To further clarify the the regulatory effect of miR-26a on PTEN, the miR-26a binding sites in PTEN were predicted using the biological prediction website. As shown in Fig. 5a, PTEN 3’UTR contained potential miR-26a binding sites. In addition, dual-luciferase reporter gene assay was performed with PTEN-Wt and PTEN-Mut co-transfected with miR-26a mimic or NC into glioma cells. Comparison with the NC mimic, the luciferase activity of PTEN-Wt was inhibited in the presence of miR-26a mimic (*p* < 0.05) (Fig. 5b), suggesting that miR-26a could specifically bind to PTEN. Furthermore, the PTEN expression in the control brain tissue and glioma tissue was quantified by RT-qPCR (Fig. 5c-d). The expression of PTEN was reduced in glioma tissues compared to the control brain tissue, and it was negatively related to miR-26a expression. Consistently, the results from the Western blot analysis in HBMECs revealed that PTEN protein level was reduced in the presence of miR-26a agomir (Fig. 5e). In contrast, PTEN protein level was increased in the presence of miR-26a antagomir, suggesting that miR-26a could suppress the PTEN expression. To further investigate whether miR-26a targets PTEN to regulate the PI3K/Akt signaling pathway in glioma, phosphorylation of proteins related to the PI3K/Akt signaling pathway was measured by Western blot analysis. As shown in Fig. 5f, p-Akt/Akt ratio was reduced in presence of PTEN, but increased in miR-26a agomir. Notably, p-Akt/Akt ratio was significantly decreased in HBMECs following the up-regulation of both miR-26a and PTEN as compared to that after the up-regulation of miR-26a alone. Based on the aforementioned results, miR-26a activated the PI3K/Akt signaling pathway through the down-regulation of PTEN.

### Exosomal miR-26a promotes proliferation and angiogenesis of HBMECs

**Fig. 6:**
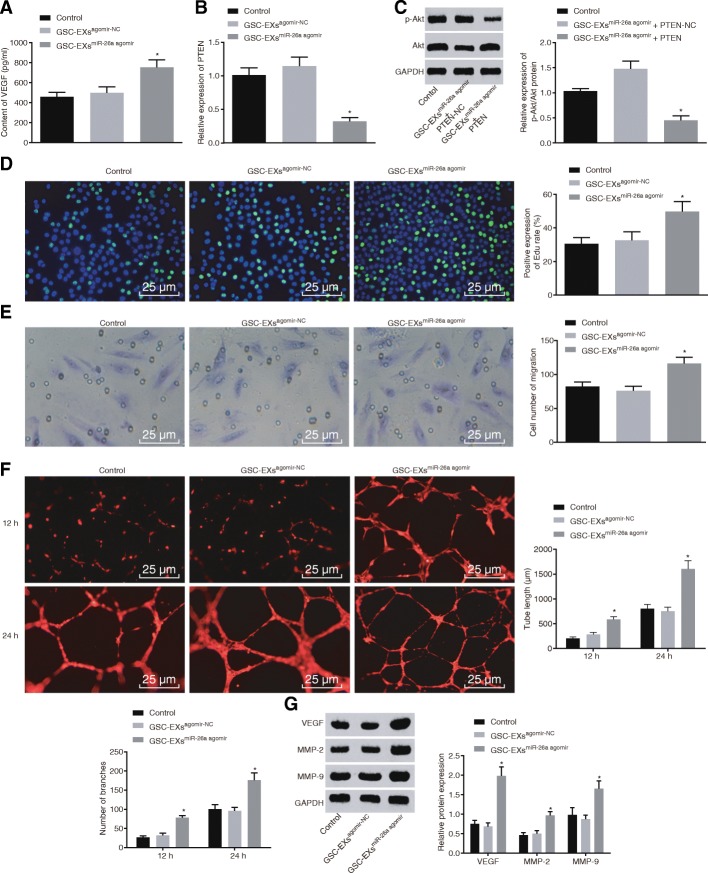
Exosomal miR-26a promotes proliferation and angiogenesis of HBMECs through the inhibition of PTEN and activation of PI3K/Akt signaling pathway. **a** VEGF level in HBMECs co-cultured with GSC-EXs^agomir-NC^ or GSC-EXs^miR-26a agomir^; **b** PTEN expression in HBMECs co-cultured with GSC-EXs^agomir-NC^ or GSC-EXs^miR-26a agomir^; **c** Akt protein level and phosphorylation level in HBMECs co-cultured with GSC-EXs^miR-26a^ + PTEN or GSC-EXs^miR-26a^ + PTEN-NC; **d** proliferation of HBMECs co-cultured with GSC-EXs^agomir-NC^ or GSC-EXs^miR-26a agomir^; **e** migration of HBMECs co-cultured with GSC-EXs^agomir-NC^ or GSC-EXs^miR-26a agomir^; **f** tube formation in HBMECs co-cultured with GSC-EXs^agomir-NC^ or GSC-EXs^miR-26a agomir^; **g** VEGF protein level in HBMECs co-cultured with GSC-EXs^agomir-NC^ or GSC-EXs^miR-26a agomir^; *, *p* < 0.05, compared with the control or GSC-EXs^agomir-NC^ group; all data were expressed as mean ± standard deviation; comparisons between two groups were analyzed using unpaired *t*-test; comparisons among multiple groups were analyzed by the one-way ANOVA; the experiment was repeated three times; ANOVA, analysis of variance; miR-26a, microRNA-26a; NC, negative control; PTEN, phosphatase and tensin homolog deleted on chromosome ten; Akt, protein kinase B; HBMECs, human brain microvascular endothelial cells; VEGF, vascular endothelial growth factor; GSC-EXs^agomir-NC^, exosomes derived from agomir-NC-transfected GSCs; GSC-EXs^miR-26a agomir^, exosomes derived from miR-26a agomir-transfected GSCs

The exosomes isolated from transfected GSCs were co-cultured with HBMECs in order to determine whether exosomal miR-26a mediates PTEN-mediated PI3K/Akt signaling pathway to regulate proliferation and angiogenesis of HBMECs. According to the ELISA analysis, VEGF level was increased in HBMECs co-cultured with exosomes overexpressing miR-26a (Fig. 6a). Moreover, RT-qPCR showed that PTEN expression was decreased in HBMECs co-cultured with exosomes overexpressing miR-26a (Fig. 6b). Consistently, the Western blot analysis revealed that restoration of PTEN caused an evident decrease in the p-Akt/Akt ratio in HBMECs co-cultured with exosomes overexpressing miR-26a (Fig. 6c). Moreover, as shown in Fig. 6d-f, HBMECs co-cultured with exosomes with overexpressed miR-26a presented with enhanced HBMEC proliferation, migration, and tube formation ability. The results from the Western blot analysis also showed that protein levels of VEGF, MMP-2, and MMP-9 were significantly increased in HBMECs co-cultured with exosomes overexpressing miR-26a (Fig. 6g). Therefore, we concluded that exosomal miR-26a inhibited the PTEN expression and activated the PI3K/Akt signaling pathway to promote proliferation and angiogenesis of HBMECs.

### miR-26a overexpression in GSCs promotes angiogenesis in vivo

**Fig. 7:**
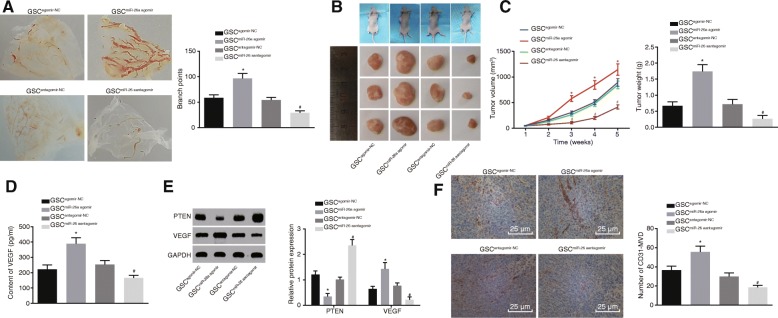
Overexpressing miR-26a in GSCs enhances angiogenesis in vivo. **a** the number of branches of blood vessels (5 mm) around the CAM vehicle; **b** transplanted tumors in nude mice; **c** tumor volume and weight; **d** VEGF level in the serum of nude mice measured by ELISA; **e** protein levels of PTEN and VEGF measured by Western blot analysis; **f** CD31-MVD detected by immunohistochemistry (× 400); *, *p* < 0.05, compared with the GSC^agomir-NC^ group; #, *p* < 0.05, compared with the GSC^antagomir-NC^ group; all data were expressed as mean ± standard deviation; comparisons between two groups were analyzed using unpaired *t*-test; comparisons among multiple groups were analyzed by the one-way ANOVA; the experiment was repeated three times; ANOVA, analysis of variance; miR-26a, microRNA-26a; NC, negative control; PTEN, phosphatase and tensin homolog deleted on chromosome ten; Akt, protein kinase B; VEGF, vascular endothelial growth factor; GSCs, glioma stem cells; CAM, chorioallantoic membrane; MVD, microvessel density; ELISA, enzyme-linked immunosorbent assay; GSC^agomir-NC^, agomir-NC-transfected GSCs; GSC^antagomir-NC^, antagomir-NC-transfected GSCs; GSC^miR-26a agomir^, miR-26a agomir-transfected GSCs; GSC^miR-26a antagomir^, miR-26a antagomir-transfected GSCs

The CAM models were established to investigate the effect of miR-26a on angiogenesis of HBMECs in vivo. As shown in Fig. 7a, the branches of blood vessels (5 mm) around the CAM vehicle were evidently increased, and the angiogenesis was enhanced in response to overexpressed miR-26a. In contrast, when miR-26a expression was inhibited, the branches of blood vessels (5 mm) around the CAM vehicle obviously reduced, and angiogenesis was inhibited. In order to further confirm that GSCs influenced angiogenesis of HBMECs in vivo, the GSCs transfected with miR-26a agomir, miR-26a antagomir and their corresponding NC plasmids were injected into the nude mice to observe the tumorigenicity and metastasis in vivo. The tumorigenicity of GSCs were tested in nude mice. The tumor size and weight were increased by enhancement of miR-26a expression, but decreased by inhibition of miR-26a (Fig. 7b-c). ELISA data (Fig. 7d) showed that there was an evident increase in serum VEGF level in the nude mice when miR-26a expression was enhanced, but significantly reduced when miR-26a expression was inhibited. Western blot analysis (Fig. 7e) showed that miR-26a overexpression led to the reduction of PTEN protein level and elevation in VEGF protein level, while these were reversed following the inhibition of miR-26a expression. Furthermore, the results from the immunohistochemistry (Fig. 7f) indicated that the angiogenesis endothelial marker, CD31-MVD value was markedly elevated in the event that miR-26a expression was increased. Taken together, the above findings demonstrated that GSCs overexpressing miR-26a could potentially enhance angiogenesis of HBMECs and tumor growth of nude mice, down-regulate PTEN expression and up-regulate VEGF expression.

## Discussion

Glioma is a type of malignant brain tumor that arises from the glial cells of the brain, ranking as one of the most commonly occurring intracranial tumors [4]. GSCs are tumor cells found in primary glioblastomas that possess stem-cell-like properties, such as self-renewal ability and further capacity to generate heterogeneous offspring [42]. It has been suggested that nanoscale vesicles, exosomes derived from GSCs served as mediators of cell-cell communication to control tumor immunity in glioma [43]. Exosomes derived from mesenchymal stem cells leads to the acceleration of angiogenesis by transferring miRNAs to endothelial cells [44]. In the present study, we showed that GSCs-exosomes overexpressing miR-26a could lead to the inhibition of PTEN and activation of the PI3K/Akt signaling pathway to promote the angiogenesis of HBMECs.

Exosomes are involved in several cellular processes including intracellular communication, material transportation on the surface of cell membrane and the regulation of cellular content during membrane fusion [45]. Exosomes derived from glioblastoma tumor cells contain intact mRNA that can be transferred to recipient cells and translated into functional proteins [46]. A study by Brower et al. demonstrated that miRNAs, which are dysregulated in malignant cells, are implicated in tumorigenesis [22]. Exosomes play a critical role in cellular communication by directly transferring genetic materials including mRNA and miRNA between cells [47, 48]. An example of this can be that the exosomes found in gliomas could deliver miR-1 to recipient cells, which then affects glioma cell invasion and proliferation as well as endothelial cell tube formation [49]. Moreover, overexpressed miR-21 in GSCs-derived exosomes can be delivered to endothelial cells (ECs) and promote the angiogenic ability of ECs [6]. Consistently, our results showed that miR-26a was up-regulated in glioma tissues and GSCs. Moreover, GSCs-derived exosomes were observed to transfer miR-26a in order to favor for elevating its expression. A recent study has reported that miR-26a contributes to enhanced angiogenesis in glioma by binding to prohibitin [23]. Moreover, miR-26a overexpression contributed to the enhanced proliferation, migration and tube formation of HBMECs. Consistent with our finding, high expression of miR-26a in glioma tissues contributes to the tumorigenesis of glioma [50]. Interestingly, miR-26a transferred from GSCs-derived exosomes into HBMECs had the ability to induce the inhibition of PTEN expression.

The results from the dual-luciferase reporter assay revealed that miR-26a targeted PTEN, and down-regulated PTEN expression. And PTEN was found to be down-regulated in GSCs as well. A previously conducted study demonstrated that miR-26a targets PTEN to promote the metastatic capacity of lung cancer cells [51]. In addition, the dysregulation of miR-26a has been reported to affect cell proliferation in glioblastoma by binding to PTEN [52]. miR-26a has also been found to be up-regulated in glioma tissues and it contributes to the tumorigenesis of glioma by targeting PTEN [50]. Consistently, our findings suggested that exosomes derived from GSCs with up-regulated miR-26a could promote proliferation, migration and tube formation of HBMECs in vitro. A previous study has indicated that miR-93 plays a regulatory role in glioma progression through the suppression of PTEN by activating the PI3K/Akt signaling pathway [53, 54]. The up-regulation of miR-26a in GSCs results in an increase in the phosphorylation of Akt, suggesting that the PI3K/AKT signaling pathway is activated by miR-26a in glioma. The aforementioned findings from our study were further supported by the findings reported by Cai et al., which demonstrated that the activation of the PI3K/Akt signaling pathway leads to the enhancement of proliferation, migration and tube formation of endothelial cells co-cultured with glioma in vitro [55].

Furthermore, our results also revealed that exosomal miR-26a derived from GSCs elevated the levels of VEGF, MMP-2 and MMP-9, thus enhancing angiogenesis of HBMECs. MMP-2 and MMP-9 are members of MMP family that is responsible for facilitating angiogenesis [56]. As a major regulator of blood vessel formation and pathological processes, VEGF modulates proliferation, migration and survival of ECs [57]. Due to its effect on EC survival, VEGF elevation triggers glioma angiogenesis [58]. Moreover, existing evidence suggests that miR-26a stimulate tumor growth and angiogenesis in glioma [23], which is consistent with our study that miR-26a in GSCs led to the enhancement of tumor growth and angiogenesis in vivo. In addition, tumor suppressor PTEN participates in regulation of tumor angiogenesis by negatively regulating PI3K [59]. The activation of PI3K/Akt signaling pathway is also associated with the acceleration of metastasis and angiogenesis in glioma [1]. The in vitro experiments confirmed our findings suggesting that GSCs-derived exosomes carrying miR-26a has the potential to enhance angiogenesis of HBMECs by inhibiting PTEN and activating the PI3K/Akt signaling pathway.

## Conclusion

**Fig. 8:**
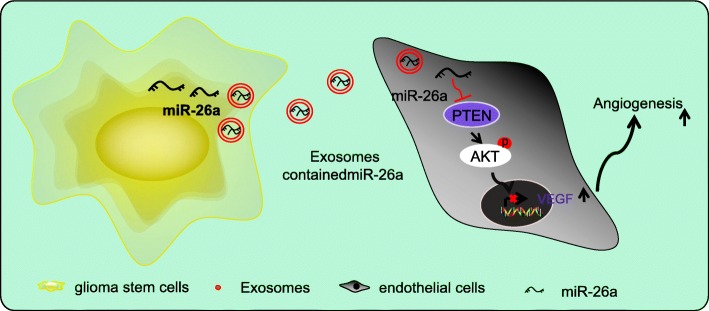
Internalization of exosomes secreted from GSCs carrying miR-26a promotes angiogenesis of HBMECs through the inhibition of PTEN and activating the PI3K/Akt signaling pathway via elevation of VEGF. PI3K, phosphatidylinositol-3 kinases; miR-26a, microRNA-26a; NC, negative control; GSCs, glioma stem cells; PTEN, phosphatase and tensin homolog deleted on chromosome ten; Akt, protein kinase B; HBMECs, human brain microvascular endothelial cells; VEGF, vascular endothelial growth factor

In conclusion, the aforementioned findings demonstrated that exosomes secreted by GSCs transferred miR-26a into HBMECs to promote the proliferation and angiogenesis of HBMECs (Fig. 8). Therefore, exosomes from GSCs with up-regulated levels of miR-26a could be potential therapeutic protocols for glioma. However, due to the fact that the research is still in the preclinical stage, the investigation on the role and mechanism of exosomal miR-26a in the angiogenesis and microenvironment of glioma in vivo is insufficient. Therefore, more experiments are required to further explore the intrinsic mechanisms of exosomal miR-26a in glioma.

## Additional file

Additional file 1: **Figure S1.** Identification of GSCs-derived exosomes. A, morphological features of GSCs-derived exosomes under the transmission electron microscope; exosomes are round or oval membranous vesicles; B, particle size of exosomes determined using Nanosight; most particles have a dimension ranged from 30 to 150 nm; C, protein bands of exosome surface markers (CD63, CD9 and CD81) detected by Western blot analysis; D, expression of exosome surface markers (CD63 and CD81) detected using flow cytometry; GSCs, glioma stem cells. (EPS 7358 kb)

## Abbreviations

ANOVA: Analysis of variance
CAM: Chicken chorioallantoic membrane
CSCs: Cancer stem cells
DEGs: Differentially expressed genes
ELISA: Enzyme-linked immunosorbent assay
FBS: Fetal bovine serum
GEO: Gene Expression Omnibus
GFP: Green fluorescent protein
GO: Gene oncology
GSC: Glioma stem cell
GSCs: Glioma stem cells
HBMECs: Human brain microvascular endothelial cells
HRP: Horseradish peroxidase
KEGG: Kyoto Encyclopedia of Genes and Genomes
MES: Morpholine-ethanesulfonate
miRNAs: MicroRNAs
MMP: Matrix metalloproteinase
Mut: Mutant
MVD: Microvessel density
MVECs: Microvascular endothelial cells
PBS: Phosphate buffer saline
PTEN: Phosphatase and tensin homolog deleted on chromosome ten
PVDF: Polyvinylidene fluoride
RIPA: Radio-immunoprecipitation assay
SD: Standard deviation
SDS: Sodium dodecyl sulfate
Wt: Wild type
